# Liver transplantation with a normothermic machine preserved fatty nonagenarian liver: A case report

**DOI:** 10.1016/j.ijscr.2019.03.033

**Published:** 2019-03-30

**Authors:** Tommaso Maria Manzia, Luca Toti, Claudia Quaranta, Francesca Blasi, Giuseppe Tisone

**Affiliations:** Department of Surgery, HPB and Transplant Unit, Tor Vergata University of Rome, Italy

**Keywords:** ALT, alanine aminotransferase, AST, aspartate aminotransferase, ECD, extended criteria donors, HCV, hepatitis C virus, LT, liver transplantation, NMP, normothermic machine perfusion, SCS, static cold storage, TACE, trans arterial chemo embolization, Liver transplantation, Extended donor criteria, Normothermic machine perfusion

## Abstract

**Introduction:**

The use of organs from expanded criteria donors for Liver Transplantation (LT) represents a major challenge. In the current era of Normothermic Machine Perfusion (NMP), donor age boundaries are often overcome and may contribute to reduce the gap between supply and demand of organs suitable for transplantation.

We report on a unique case of nonagenarian liver successfully transplanted after NMP.

**Presentation of case:**

A fatty previously declined liver graft from nonagenarian male brain death donor underwent NMP (OrganOx Metra^®^, UK, Oxford) perfusion at University of Rome Tor Vergata on April 2018. The histology assessment showed 15% macro and 35% micro vescicular steatosis. Liver fulfilled viability criteria after NMP and was thus transplanted. The recipient was a 53 years old male with hepatocellular carcinoma who underwent two previous trans-arterial chemo-embolization. The post-LT AST peak was 1556 U/L; post operative course was characterized by hepatic artery thrombosis that required re-laparotomy and successful thrombectomy. Recipient experienced biliary stricture three months after discharge successfully treated by endoscopic retrograde cholangiopancreatography. At 7 months of follow-up patient has good clinical status and graft function.

**Discussion:**

NMP represents a safe approach in order to increase the usage of very old fatty livers, that otherwise would be declined because of the high risk of primary non function and death.

**Conclusion:**

Nonagenarian liver recruitment after NMP seems to be feasible but a major attention is advisable on the manipulation and cannulation of hepatic artery in order to avoid intimal damage that can lead hepatic artery thrombosis.

## Highlights

• The ex-situ normothermic machine perfusion provide a successful recondition of nonagenarian liver.
• Gentle cannulation of hepatic artery is mandatory in order to avoid unwanted intimal damage.
• In order to minimise post LT hepatic artery thrombosis jeopardy no further risk, as previous multi-trans arterial chemoembolization should be taken.

## 1 Introduction

Liver transplantation (LT) is the standard of care in patients with end-stage liver diseases [1]. Nowadays, because of donor shortage, about 10% of potential liver transplant recipients still die on the waiting list [2]. In order to increase the potential donor pool, the use of expanded criteria donors became a widespread accepted strategy. However, the extended criteria livers suffer from ischaemic-reperfusion injury due to static cold storage (SCS) with subsequent higher risk of graft failure, complications and mortality [3]. The ex situ normothermic machine perfusion (NMP) is a device based on dynamic liver preservation with oxygenated blood, nutrients and medications at normal body temperature, which has the purpose to maintain the graft functioning and physiological status, thus avoiding ischaemic injury caused by SCS [4]. We report a single and unique case of a nonagenarian liver recruited and successfully transplanted at Tor Vergata University, Rome, Italy after NMP. The current work has been reported in line with SCARE Criteria [5].

## 2 Presentation of case

In April 2018, a 88 years old male brain death donor was declined by all local transplant centres due to high-risk of graft loss. At the time of procurement the reasons for discarding liver were elderly age and macroscopic appearance of moderate firmness, round liver edges and suboptimal liver graft perfusion. The liver histology findings detected 15% macro and 35% micro-vesicular steatosis. Major donor comorbidities included hypertension, hypertensive heart disease and chronic obstructive pulmonary syndrome. The donor liver function tests were in normal range, and the inotropic support was 1 × 10^−7^ g/kg BW/min. The donor risk Index was 2: considering the donor age as well as both surgical appearance and histological findings, liver was considered not eligible for transplantation by itself and was connected to the NMP (OrganOx Metra^®^, Oxford, UK) at the donor hospital.

Haemodynamic [hepatic artery flow (>0.150 ml/min) and portal vein flow (>0.500 ml/min)] and metabolic parameters [pH > 7.3; lactate clearance (<2.5 within 4 h) and bile production] [6] were evaluated in order to address viability. The current case and all extended criteria donors (ECD) considered for NMP in our centre met the viability assessment and the criteria approved by our local ethical committee (15th January 2018, Registro Sperimentazioni n: 214/17, Fondazione PTV).

**Fig. 1:**
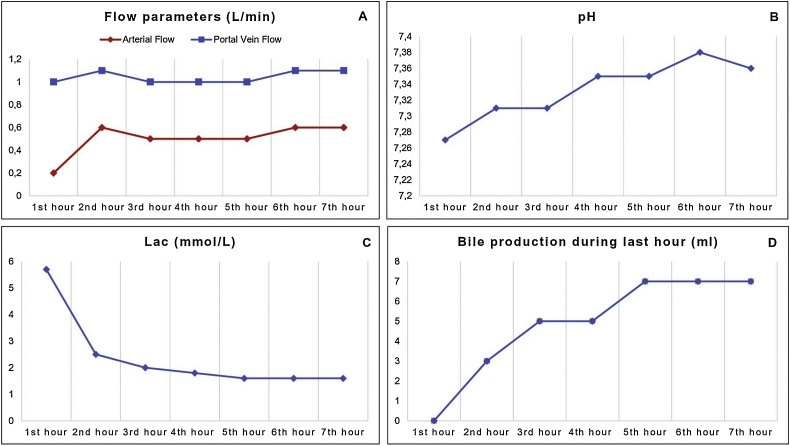
Liver functions parameters during normothermic machine perfusion: (A) Hepatic artery and portal vein flow rate; (B) pH; (C) lactate clearance; (D) bile production.

At the beginning of the perfusion, the hepatic artery and portal vein flow rate were 0.200 ml/min and 1000 ml/min respectively and remained stable throughout the perfusion time (Fig. 1A). The pH at start was 7.27 and reached normal range within 2 h (Fig. 1B); lactate dropped from 5.7 to 1.6 mmol/L before implantation (Fig. 1C). A total amount of 23 g of bile was produced (Fig. 1D). Perfusate alanine aminotransferase (ALT) and aspartate aminotransferase (AST) reached a peak of 1386 U/L and 1170 U/L respectively.

After a perfusion of 282 min, the graft fulfilled all viability criteria [6] thus was considered suitable for transplantation.

The recipient was a 53 years old caucasian male with Hepatocellular Carcinoma fulfilling Milan criteria after two trans arterial chemo embolization (TACE) with underlined HCV- cirrhosis (biochemical MELD 7) who consented for NMP; alpha-fetoprotein (AFP) level at listing was 5.72 UI/ml, balance of risk (BAR) [7] was 4. LT was performed at Tor Vergata University, Rome Italy by highly experienced liver transplant surgeon using Piggyback technique; arterial anastomosis was performed using 6/0 prolene running suture between recipient proper hepatic artery and donor common hepatic artery.

After the hepatectomy, the liver was disconnected from the NMP and flushed with 2 l of cold Celsior^®^ Solution; total perfusion time in NMP was 478 min. The mean arterial pressure, vasopressors support [8] and serum kalium levels during and after liver reperfusion are reported in Table 1.

**Table 1:** Ischemia Reperfusion Injury parameters: mean arterial pressure, norepinephrine infusion and serum kalium levels before and after liver reperfusion.

	Mean arterial pressure MAP (mmHg)	Norepinephrine infusion NE (g/KgBW/min)	Serum kalium levels (mmol/L)
Initiation of surgery	90	1 × 10^−7^	3.7
Anhepatic phase	80	1 × 10^−7^	3.1
Before graft reperfusion	80	1 × 10^−7^	3.4
5 min after graft reperfusion	80	1 × 10^−7^	3.1
30 min after graft reperfusion	83	1 × 10^−7^	3.1
60 min after graft reperfusion	83	1 × 10^−7^	3.1
90 min after graft reperfusion	83	1 × 10^−7^	3.5

**Fig. 2:**
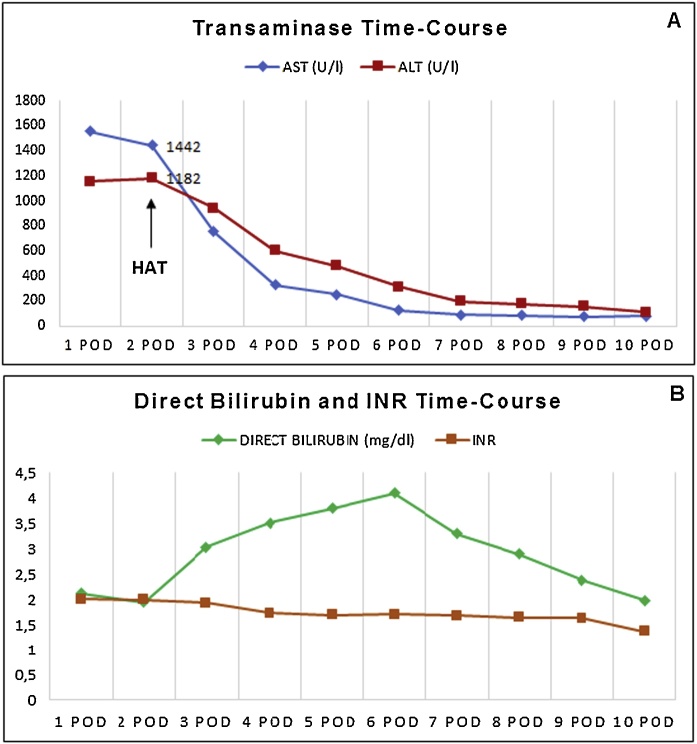
Recipient liver function tests within 10 post operative days: (A) ALT, alanine aminotransferase; AST, aspartate aminotransferase; (B) Bilirubin and Prothrombin time (PT-INR).

The macroscopic appearance after reperfusion was excellent and intraoperative ultrasound showed good artery and portal flow. AST and ALT time-course as well as bilirubin and INR trends during the post-operative days are displayed in Fig. 2. Intensive Care Unit stay was 72 h. Notably, post-operative course was complicated by hepatic artery thrombosis (HAT) detected with Doppler ultrasound performed at 2nd post-operative days that required a re-laparotomy and a successful thrombectomy. The patient was discharged on 9th postoperative day with good clinical status and graft function.

Three months after LT the patient suffered a non-anastomotic biliary stricture that required endoscopic retrograde cholangiopancreatography with stent positioning. At last follow up (7 months) patient showed good clinical status and graft function.

## 3 Discussion

Between December 2017 and September 2018, in Italy out of 1360 donors suitable for donation 1201 livers were eligible for transplantation [2]; this resulted in 12% of discard rate during multi-organ retrieval (n: 159 livers); data also in accordance with UK Transplant Registry [9] where out of 1488 liver retrieved only 85% were finally transplanted. Importantly, the DCD activity in Italy is still at the early stages and all donors considered are from DBD. In West worldwide countries most of cadaveric donors are considered as extended criteria [10,11] often allocated, in order to reduce post-transplant morbidity and mortality, to low-risk recipients, thus limiting the access to LT to high risk patients. The machine-based perfusion techniques represents one of the promising strategies in order to decrease the gap between the demand and organs supply. NMP has proven to be feasible, safe and effective to predict the liver viability either in standard or extended donor. Recently the Birmingham Group demonstrated as NMP (OrganOx Metra^®^, Oxford, UK) increased the utilization rate of 50% vs SCS liver, decreasing also early allograft dysfunction and AST peak [4]. In 2016 Mergental et al reported the first clinical series of five successfully transplanted liver retrieved from declined ECD liver in UK [12] and most recently He et al. [13] described the very first 85–95% macrovescicular steatotic liver successfully transplanted after ischemic-free normothermic preservation. Considering the experience achieved worldwide, Porte RJ Group [14] recently published a snapshots of potentially viability tests for hepato/cholangio-cellular functions (i.e. bile production, perfusate glucose and lactate; Bile pH) or injury (perfusate aminotrasferase) in order to best define the organs suitable for transplantation and possibility to rehabilitate high risk livers with glucose bolus, bicarbonate, oxygen [15] and perhaps in next near future with statins [16] also; this could result in increasing suitable organs with decreasing recipients mortality risks. The temperature (i.e. cold; normothermic or sub-normothermic) as well as the type of fluid to flush out all waste products during the NMP still remains an unanswered question; in this case, as local policy we flushed out the liver by a cold solution used also for procurement.

The viability assessment has been mostly adopted in a range adult age between 16 and 84 years old (COPE trial) [4]. Therefore, to the best of our knowledge, the current case represents the first ninety implanted liver described so far. The hepatocellular and haemodynamics assessment taken during perfusion fulfilled all criteria described in 2017 by Laing in a VITTAL protocol [7] (namely perfusate lactate <2.5 mmol/L; pH > 7.3; evidence of bile production; stable portal and arterial flow; homogeneous perfusion). Unfortunately, patient experienced early HAT that has been successfully treated by surgical thrombectomy. Although the correlation between NMP and HAT is difficult to ascertain, we can hypothesize that the association between arteriosclerosis from the old donor, manipulation of the graft hepatic artery (by cannulation and continuous perfusion) and the two previous upfront TACE in the recipients could lead intimal damage and shear stress causing arterial thrombosis. Even if the recipient at last follow up, did completely recover, only further close clinical follow up will detect any potentially life-threating biliary ischemic disease.

Due to the poor experience and the paucity of data available in the literature, we decided to allocate this “high risk donor” to a well-compensated recipient (BAR:4) in order to decrease the post-operative risk; however, we can maybe argue that a well-reconditioned extended nonagenarian liver that fulfil viability-criteria could be even allocated to a higher risk recipient. This could be an option in order to minimize the waiting list time in an organ-shortage era. The main learning point of this single case is that a great caution must be taken on the artery cannulation and perfusion of these very old donors and no recipient further risk, as previous TACE, should be taken in order to avoid unwanted arterial complications. Randomized controlled trial powered only for HAT in elderly donors assessed with NMP are needed in order to well define the HAT risk after LT.

## Conflicts of interest

The authors of this manuscript have no conflicts of interest to disclose, as described by *International Journal Surgery Case* Report.

## Sources of funding

This research did not receive any specific grant from funding agencies in the public, commercial, or not-for-profit sectors.

## Ethical approval

The current case as well as all extended criteria donors (ECD) considered for NMP in our centre met the viability assessment and the criteria approved by our local ethical committee (15th January 2018, Registro Sperimentazioni n: 214/17, Fondazione PTV).

## Consent

Written informed consent was obtained from the patient for publication of this case report. A copy of the written consent is available for review by the Editor-in-Chief of this journal on request

## Author contribution

Tommaso Maria Manzia wrote the manuscript. Luca Toti, Claudia Quaranta and Francesca Blasi collected and analyzed data. Giuseppe Tisone has approved the final version

## Registration of research studies

NA.

## Guarantor

Prof Giuseppe Tisone.

## Provenance and peer review

Not commissioned, externally peer-reviewed.
